# Genetic defects in SAPK signalling, chromatin regulation, vesicle transport and CoA-related lipid metabolism are rescued by rapamycin in fission yeast

**DOI:** 10.1098/rsob.170261

**Published:** 2018-03-28

**Authors:** Kenichi Sajiki, Yuria Tahara, Alejandro Villar-Briones, Tomáš Pluskal, Takayuki Teruya, Ayaka Mori, Mitsuko Hatanaka, Masahiro Ebe, Takahiro Nakamura, Keita Aoki, Yukinobu Nakaseko, Mitsuhiro Yanagida

**Affiliations:** 1 Okinawa Institute of Science and Technology Graduate University, 1919-1 Tancha, Onna-son, Okinawa 904-0495, Japan; 2 Graduate School of Biostudies, Kyoto University, Sakyo-ku, Kyoto 606-8501, Japan

**Keywords:** rapamycin, SAPK, fission yeast, mutant screening, quantitative metabolomics

## Abstract

Rapamycin inhibits TOR (target of rapamycin) kinase, and is being used clinically to treat various diseases ranging from cancers to fibrodysplasia ossificans progressiva. To understand rapamycin mechanisms of action more comprehensively, 1014 temperature-sensitive (ts) fission yeast (*Schizosaccharomyces pombe*) mutants were screened in order to isolate strains in which the ts phenotype was rescued by rapamycin. Rapamycin-rescued 45 strains, among which 12 genes responsible for temperature sensitivity were identified. These genes are involved in stress-activated protein kinase (SAPK) signalling, chromatin regulation, vesicle transport, and CoA- and mevalonate-related lipid metabolism. Subsequent metabolome analyses revealed that rapamycin upregulated stress-responsive metabolites, while it downregulated purine biosynthesis intermediates and nucleotide derivatives. Rapamycin alleviated abnormalities in cell growth and cell division caused by *sty1* mutants (Δ*sty1*) of SAPK. Notably, in Δ*sty1*, rapamycin reduced greater than 75% of overproduced metabolites (greater than 2× WT), like purine biosynthesis intermediates and nucleotide derivatives, to WT levels. This suggests that these compounds may be the points at which the SAPK/TOR balance regulates continuous cell proliferation. Rapamycin might be therapeutically useful for specific defects of these gene functions.

## 1. Introduction

Rapamycin is an antifungal metabolite discovered in the 1970s in *Streptomycin hygroscopicus*. It inhibits cell proliferation and antibody formation by T cells, which prompted its use as an immunosuppressive drug for organ transplantation (known by the generic name Sirolimus) [1]. Rapamycin forms a complex with peptidyl-prolyl-isomerase, FKBP12, to inhibit serine/threonine protein kinases designated TOR (target of rapamycin) [2–5]. TOR kinases comprise two distinct protein complexes inside cells that regulate cell proliferation [6]. Various studies have shown that many TOR pathway components are associated with cancer [7–10], and as a result, rapamycin has been used as an anti-tumour drug. Clinical trials have confirmed its effectiveness against mammary tumours, colon cancer, melanocarcinoma and ependymoblastoma [11]. In addition, rapamycin also extends the lifespans of yeast, fruit flies and mice [12–15], probably by reducing calorie consumption. Recently, rapamycin became the first drug in clinical trials for the treatment of fibrodysplasia ossificans progressiva, using patient-derived induced pluripotent stem cells [16]. Most recently, rapamycin has also proved effective in treating Pompe disease, which causes lysosomal glycogen accumulation in skeletal muscle and heart [17].

The fission yeast *Schizosaccharomyces pombe* has proved to be an excellent model for studying cellular functions of rapamycin and the TOR pathway, owing to the availability of genetic methods, comprehensive mutant libraries and high conservation of mammalian TOR pathway components [18]. In our previous report, we discovered that temperature sensitivity (ts) of *cut1*/separase and *cut2*/securin mutants was rescued by rapamycin, illuminating a new aspect of TOR signalling in cell growth and division [19]. This discovery suggested that there still exist undiscovered ts mutants that could offer a more comprehensive understanding of rapamycin. Accordingly, in the hope of discovering novel applications of this drug, we tested the capability of rapamycin to rescue mutants in our ts mutant library, which contains 1014 strains [20]. Mutants defective in 12 genes were found to be rescued by rapamycin. Among them, *sty1* mutants were further studied to show that rapamycin alleviated abnormal growth and division of these mutants. Also, we report a metabolome analysis to reveal the cellular impact of rapamycin. Metabolomic results strongly suggested that purine biosynthesis is implicated in the critical regulation targeted by rapamycin.

## 2. Results

### 2.1. Screening of the ts mutant library for rapamycin rescue

**Figure 1.**
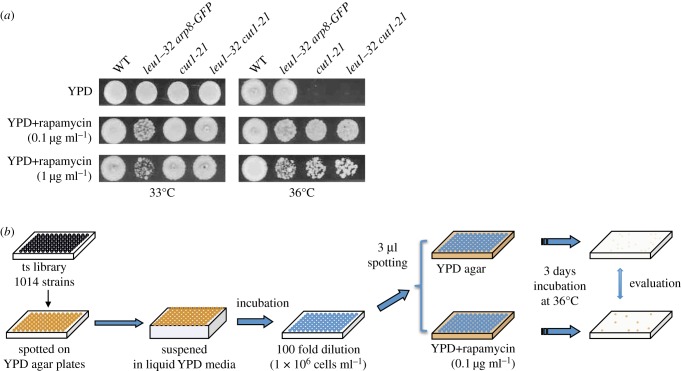
Screening of 1014 temperature-sensitive mutants yielded 45 strains in which the ts phenotype was rescued by rapamycin. (*a*) Spot tests of WT (h^−^ 972), parental strain (*leu1–32 arp8-GFP*), the previously reported *cut1-21* strain, on YPD plates with 0.1 µg ml^−1^ rapamycin at 36°C clearly showed the rescue effect. (*b*) Schematized procedures of bulk screening using 96-well plates.

**Figure 2.**
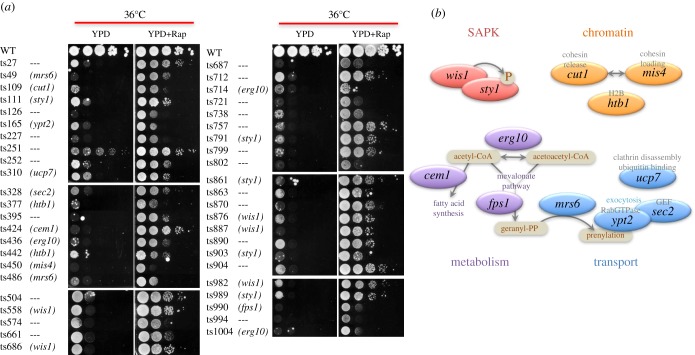
Twelve genes were responsible for the ts phenotypes of strains identified as responsive to rapamycin. (*a*) Spot tests of 45 strains on YPD or YPD + rapamycin (Rap) plates at 36°C. Names of responsible genes are in parentheses next to strain numbers. (*b*) The 12 responsible genes were classified into four groups, based on their reported cellular functions.

For library screening, a pilot spot test using a control strain (*cut1-21*) [19] showed that a rapamycin concentration of 0.1 µg ml^−1^ was useful (figure 1*a*), because higher concentrations (e.g. greater than 1 µg ml^−1^) inhibited growth of the parental strain of this library (*leu1-32 arp8-GFP*) at the restrictive temperature (36°C). Therefore, using an automated robot system, screening was conducted by spotting 1014 ts mutants [20] onto YPD plates with or without 0.1 µg ml^−1^ rapamycin (figure 1*b*). At 36°C, 62 strains showed better colony formation on YPD plates with rapamycin than without (electronic supplementary material, figure S1). These strains were further tested by serial dilution and manual spotting on plates to eliminate false positives (electronic supplementary material, figure S2). In 45 strains, it was confirmed that rapamycin rescued the ts phenotypes (figure 2*a*). Then, the mutated genes responsible for ts sensitivity were identified by suppression analysis and by either tetrad analysis or whole-genome sequencing (electronic supplementary material, table S1, Material and methods).

### 2.2. Rapamycin-rescued ts phenotypes of mutations in 12 genes

Among these 45 strains, 12 genes proved responsible for the ts phenotype (electronic supplementary material, table S1). They must be sole responsible genes for each strain, because plasmids containing WT genes for other background mutations could not rescue the ts phenotype. Based on reported functions, they belonged to four categories (figure 2*b*): stress-activated protein kinases (SAPK) (two genes), chromatin regulation (three genes), transport (four genes) and lipid metabolism (three genes).

The largest number of strains involved either of two SAPK mutants, *sty1* (MAPK; five strains) and *wis1* (MAPKK; five strains) (figure 2*a*). These genes are central to the stress-responsive signalling pathway, and Wis1 phosphorylates Sty1 [21,22]. Mutations in all *sty1* and *wis1* strains with amino acid substitutions were located in or near their protein kinase domains, and all with nonsense mutations (*sty1-791*, *wis1-887*, *sty1-989*) lacked the C-terminal half of the kinase domains (electronic supplementary material, table S1). Given their abundance, we examined these mutants further.

In the chromatin regulation gene group, one *cut1* mutant (*cut1-109*) was identified in addition to the previously reported *cut1-21* [19]. Cut1 cleaves Rad21, which is a subunit of cohesin complex. It should be noted that 18 other *cut1* mutants exist in this library, so further spot tests were performed for them. In fact, most of them revealed a more temperature-sensitive phenotype, which was also rescued by rapamycin (electronic supplementary material, figure S3A). Mutation sites were concentrated in Cut1's peptidase and central domains (electronic supplementary material, figure S3B). Other chromatin regulation genes included *mis4*, a cohesion loader for establishing sister chromatid cohesion [23,24], and *htb1*, a histone H2B (figure 2*c*). Four other *htb1* mutants have been identified in this library. Of those, two were mutated at the N-terminus: *htb1-377* (G30D) and *htb1-442* (E35 K) and showed the rescue phenotype, while *htb1-72* (G52D) and *htb1-223* (P102 L) did not [25]. The N-terminal end of H2B is phosphorylated under stressful conditions [26,27], suggesting the basis for this allelic difference.

In the vesicle transport gene group, one clathrin-binding gene (*ucp7*), one exocytic Rab-type GTPase gene (*ypt2*) and its GTP exchange factor (*sec2*), and one Rab geranylgeranyl transferase-related gene (*mrs6*) were identified (figure 2*b*).

In the metabolism gene group, there were three genes related to CoA metabolism and the mevalonate pathway: *erg10* (acetyl-CoA C-acetyltransferase), *cem1* (3-oxoacyl-[acyl-carrier-protein]-synthase condensing enzyme) and *fps1* (farnesyl diphosphate synthase) (figure 2*b*).

### 2.3. Rapamycin-induced cell division at 36°C, in contrast to SAPK mutations

**Figure 3.**
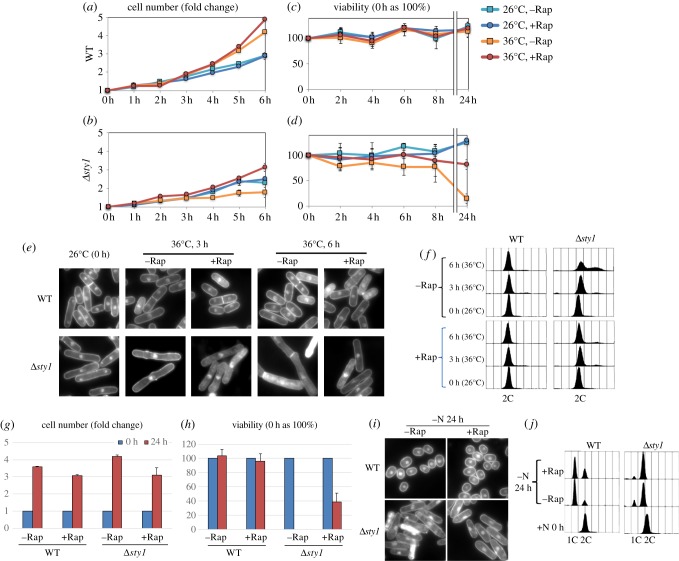
Rapamycin rescued abnormal cell division, growth and DNA content in Δ*sty1* mutants. (*a*–*d*) Cell number increment and viability of WT and Δ*sty1* in EMM2 liquid medium with or without 200 nM rapamycin at 26°C or 36°C were plotted in a time course. Error bars showed standard deviation. (*e*) DAPI images of WT and Δ*sty1* cells under the conditions indicated. (*f*) DNA content of WT and Δ*sty1* cells under the indicated conditions. (*g*,*h*) Cell number increment (*g*) and viability (*h*) of WT and Δ*sty1* before and 24 h after nitrogen deprivation (−N) in media with or without 200 nM rapamycin. Error bars showed standard deviation. (*i*) DAPI images of WT and Δ*sty1* cells at 24 h after −N in media with or without 200 nM rapamycin. (*j*) DNA content of WT and Δ*sty1* before and 24 h after −N in media with or without 200 nM rapamycin.

All SAPK ts mutants showed identical phenotypes, and we also found a deletion strain of *sty1* (Δ*sty1*) that was similar. As with ts mutants, the Δ*sty1* strain showed reduced viability at 36°C, which was rescued by rapamycin (figure 3*d*). Therefore, Δ*sty1* was used to study functional implications of SAPK and rapamycin, in order to eliminate potential dominant negative effects of ts mutants.

At the permissive temperature, 26°C, Δ*sty1* cells multiply slightly more slowly than WT, displaying longer cell lengths (figure 3*a*,*b*,*e*). At 26°C, addition of 200 nM rapamycin did not have a significant effect upon the rate of cell division, a negative result reported previously [28]. At 36°C, Δ*sty1* showed a ts phenotype with retarded mitotic increase, while WT cells divided much more rapidly (figure 3*a*,*b*). At this temperature, rapamycin rescued the ts phenotype of Δ*sty1*, accelerating cell division approximately 1.63-fold (figure 3*b*). Similar effects were also observed in the ts strain, *sty1-989* (electronic supplementary material, figure S4).

### 2.4. Rapamycin-rescued nuclear abnormality and viability loss of the SAPK mutant, Δ*sty1*, at 36°C, even under nitrogen deprivation

Cell morphology of Δ*sty1* was further examined at 36°C. Six hours after the temperature shift, cell size was abnormally elongated and septated cells were frequently observed (figure 3*e*). Also, nuclei appeared enlarged and deformed in Δ*sty1*. Such deformation was moderated by rapamycin. In fact, subsequent FACS analysis of Δ*sty1* showed that its 2C DNA peak expanded to the right at 36°C, but this expansion was alleviated when rapamycin was added (figure 3*f*). Thus, Sty1 and rapamycin may maintain proper nuclear and cell shapes at 36°C.

Previously, we reported that SAPK mutants showed abnormally expanded nuclei and severely decreased viability after nitrogen deprivation (−N) [29], so we tested whether rapamycin could also rescue this phenotype. After −N, WT cells undergo two divisions without cell growth, resulting in a roughly fourfold increase in cell number. G0 phase cells are small and round, and most have 1C DNA content (figure 3*g*,*i*,*j*). They complete G0 phase entry within 24 h after −N, and maintain high viability (figure 3*h*). However, Δ*sty1* mutant cells could not stop cell growth or division after −N, increasing in number by about 4.3-fold. Cells were elongated with 2C DNA content (figure 3*g*,*i*,*j*). This demonstrates a clear failure of G0 phase entry, and cells lost all viability 24 h after −N (figure 3*h*), as with ts mutants reported previously [29]. When rapamycin was added to *Δsty1*, however, cell number increment after −N was suppressed to the WT level, and viability recovered by 39% (figure 3*g*,*h*). Interestingly, this rescue occurred without rescuing the elongated cell shape and 2C DNA content (figure 3*i*,*j*), implying cell morphology and viability are separately regulated under −N.

### 2.5. Rapamycin induces stress-responsive metabolites and basic amino acid derivatives at 36°C

**Figure 4.**
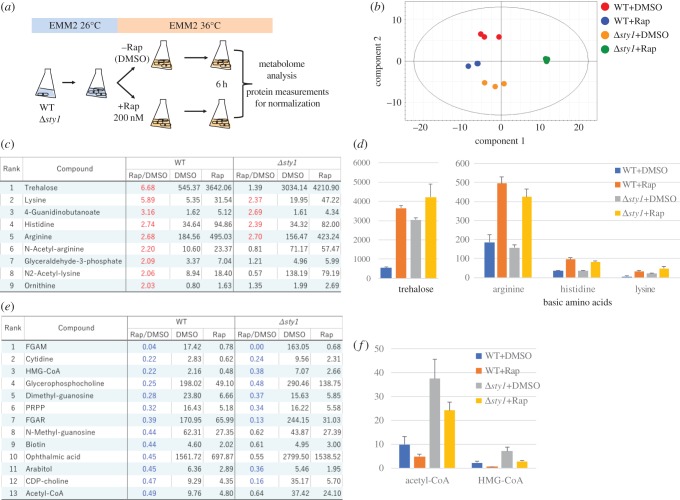
Metabolome analysis revealed the cellular impact of rapamycin in WT and Δ*sty1*. (*a*) Schematized procedures of metabolome analysis. (*b*) Principal components analysis (PCA) was conducted for the entire dataset of 98 metabolites (electronic supplementary material, table S2). A scatter plot of scores of the two principal components was shown. (*c*) List of metabolites that increased greater than twofold in a WT sample with rapamycin (Rap) compared with a control DMSO sample (DMSO). The DMSO and Rap columns show normalized peak areas and Rap/DMSO column shows the fold change. (*d*) Normalized mean peak areas with standard deviations of trehalose and basic amino acids in WT and Δ*sty1* with DMSO or Rap. (*e*) List of metabolites that decreased more than 50% in a WT sample with Rap compared with DMSO. (*f*) Normalized mean peak areas with standard deviations of acetyl-CoA and HMG-CoA in WT and Δ*sty1* with DMSO or Rap.

In order to examine effects on metabolism induced by rapamycin, we conducted quantitative metabolomic analysis of WT and Δ*sty1* cell cultures incubated for 6 h at 36°C with or without 200 nM rapamycin, the smallest concentration able to inhibit TOR signalling [28] (figure 4*a*). Since cell size varied among strains and conditions, data were normalized by protein concentration (electronic supplementary material, table S2). The experiment was run in triplicate and reproducibility was confirmed using principal components analysis (figure 4*b*).

In WT, compared with DMSO (the solvent used for rapamycin, as a control), rapamycin increased nine metabolites greater than twofold (figure 4*c*). Those metabolites were trehalose, basic amino acids and their acetylated forms (arginine, histidine, lysine, *N*-acetyl-arginine, *N*-acetyl-lysine, 4-guanidinobutanoate, ornithine), and glyceraldehyde-3-phosphate. Trehalose, which showed the greatest increase, is well known as a stress-responsive metabolite [30]. Interestingly, it was also significantly induced in WT by rapamycin addition, but in Δ*sty1*, the trehalose level was already high before rapamycin addition (figure 4*d*), implying that Sty1 might control stress response before adding rapamycin. WT and Δ*sty1* both increased basic amino acid titres after rapamycin addition (figure 4*d*).

### 2.6. Rapamycin reduces purine biosynthetic intermediates, nucleotide derivatives and CoA related metabolites at 36°C

In WT, rapamycin caused 13 metabolites to decrease to less than half (figure 4*e*). The majority of these were purine biosynthesis intermediates (FGAM, FGAR, PRPP) and nucleotide-related metabolites (cytidine, mimethyl-guanosine, *N*-methylguanosine, CDP-choline). They accounted for more than half of the decreased metabolites, implying that nucleotide metabolism is a major target of rapamycin.

Beside nucleotide derivatives, HMG-CoA and acetyl-CoA decreased by 78% and 51%, respectively (figure 4*f*). As mentioned above, another major group of mutants rescued by rapamycin is involved in CoA metabolism (figure 2*b*). Inhibition of the CoA pathway by rapamycin may be the basis for rescuing such mutants.

### 2.7. In the SAPK mutant, Δ*sty1*, rapamycin abolished overproduction of purine biosynthesis intermediates and nucleotide derivatives

**Figure 5.**
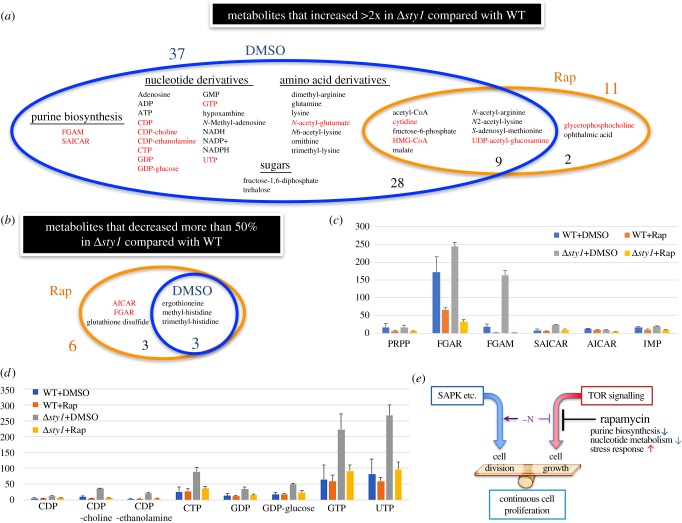
Rapamycin abolished the overproduction of most metabolites in Δ*sty1*. (*a*) Venn diagram of metabolites that increased greater than twofold in Δ*sty1* compared with WT with DMSO or Rap. In the presence of rapamycin, metabolites (in red) decreased to less than 50% of their values when Δ*sty1* was cultured in DMSO. (*b*) Venn diagram of metabolites that decreased more than 50% in Δ*sty1* compared to WT with DMSO or Rap. In the presence of rapamycin, metabolites (in red) decreased to less than 50% of their values when Δ*sty1* was cultured in DMSO. (*c*,*d*) Normalized mean peak areas with standard deviations of purine biosynthesis intermediates (*c*) and nucleotide derivatives (*d*) in WT and Δ*sty1* with DMSO or Rap. (*e*) Model of the relationship between identified genes like SAPK and TOR signalling for cell growth and division. If growth and division are well balanced, continuous cell proliferation is maintained. However, if division becomes excessive, as in −N conditions, after several mitotic divisions, continuous proliferation is abandoned. Twelve genes identified in this study may be important for maintaining the appropriate balance.

Metabolomic results indicated that many metabolites were overproduced in Δ*sty1* cells. In those cells, 37 metabolites were found at concentrations greater than twofold the level seen in WT cells (figure 5*a*, DMSO; electronic supplementary material, table S2). However, with rapamycin addition, most of these overproduced metabolites returned to WT levels, and only 11 remained greater than twofold (figure 5*a*, Rap; electronic supplementary material, table S2). Among those adjusted to WT level by rapamycin, those that decreased more than 50% included biosynthesis intermediates (FGAM, SAICAR; figure 5*c*) and nucleotide derivatives (CDP, CDP-choline, CDP-ethanolamine, CTP, GDP, GDP-glucose, GTP, UTP; figure 5*d*). This implies that purine biosynthesis and nucleotide metabolism might be key regulatory targets of SAPK and rapamycin.

On the other hand, only three metabolites in Δ*sty1* decreased to less than 50% of the WT (figure 5*b*, DMSO; electronic supplementary material, table S2). Those were ergothioneine and methylated histidines, which could be related to stress response. When rapamycin was added, three more metabolites in *Δsty1* decreased to less than 50% of the WT (figure 5*b*, Rap; electronic supplementary material, table S2). Again, two were purine biosynthesis intermediates (AICAR, FGAR), further suggesting that the purine pathway could be rapamycin's physiological target.

## 3. Discussion

In this study, we identified mutants in which the ts phenotype was rescued by rapamycin. Although roles of TOR have been reported in diverse cellular processes from protein synthesis to autophagy [6], the 12 genes identified here were rather narrowly focused.

The most abundant were SAPK mutants, *wis1* and *sty1*, each comprising five ts alleles. Four *wis1* and three *sty1* mutants involved single amino acid substitutions. Further, all but one (*wis1-982*) of these seven missense mutations occurred at conserved amino acids in the catalytic domains of Wis1 and Sty1, strongly suggesting that the loss of function for these protein kinases might be compensated by rapamycin-dependent inactivation of TOR signalling. Thus, the TOR and SAPK signalling pathways seem most likely to have opposing principal cellular functions.

The most striking aspect of suppression of the SAPK Δ*sty1* mutant by rapamycin is that cell viability is restored in both +N and −N media when rapamycin is added (figure 3*d*,*h*), whereas the abnormal rod-like shape of Δ*sty1* cells remains in −N media (figure 3*i*). Furthermore, the DNA content of Δ*sty1* in −N media remains 2C (figure 3*j*), indicating that rapamycin cannot restore G0 phase quiescent cell shape and DNA content. It is thus critically important to determine the rapamycin target for restoring cell viability.

The mechanism of rescue required further investigation. One possibility was suggested by our metabolome analysis, showing that overproduction of purine biosynthesis intermediates and nucleotide derivatives in Δ*sty1* was abolished by rapamycin. In excess, these metabolites in Δ*sty1* cells might cause cell death. Rapamycin especially reduced the level of purine intermediates, like FGAM. In support of such a conclusion, mTORC1 was recently reported to be linked to de novo purine synthesis via the mitochondrial tetrahydrofolate cycle [31]. These are just our initial findings for complex regulation between SAPK and TORC, and we would like to continue more detailed metabolome analyses in future to reveal the mechanisms.

In addition to SAPK, we also identified chromatin regulation, vesicle transport and CoA metabolism genes from rapamycin screening. The chromatin regulation group comprised three genes rescued by rapamycin. Among them, Cut1 and Mis4 are for cohesin release and loading of chromatin, respectively [23,24,32,33], while Htb1 is for nucleosomal histone H2B function [25]. Previously, a possible explanation for the rescue of *cut1* mutants by rapamycin was thought to be that the crucial balance between TORC1 and Cut1/separase might prevent premature sister chromatid separation, in which low TORC1 activity alleviates the Cut1 requirement [19]. However, it may be that in these mutants, the delicate balance between cell elongation and cell division is modulated by rapamycin through its influence over TORC signalling responding to nutritional cues.

The vesicle transport group comprised four genes. Regulation of endosome maturation and endocytosis/exocytosis by TOR signalling and/or rapamycin has been reported [34–38], so some strains rescued by inhibiting the TOR pathway may involve this mechanism.

The metabolism group was related to lipid metabolism and CoA regulation, comprising three genes, Erg10, Fps1 and Cem1. Erg10 catalyses reactions between acetyl-CoA and acetoacetyl-CoA [39]. Both are substrates for the mevalonate pathway to produce geranyl diphosphates for prenylation of Rheb and Rab GTPases [40–42] (figure 2*c*). Lipids are required for homeostatic synthesis of new membranes during G0 quiescence. TOR signalling is reported to activate the mevalonate pathway via the sterol-responsive element-binding protein (SREBP) [43,44]. Notably, because *mrs6* (Rab geranylgeranyl transferase) was also identified in addition to *fps1*, these metabolism genes could contribute to prenylation of vesicle transport proteins. Cem1 is a beta-keto-acyl synthase that directs the use of acetyl-CoA in fatty acid biosynthesis [45]. These metabolism genes may also work to balance synthesis and degradation of lipids.

Previously, large-scale screening of yeast deletion libraries has been conducted to test rapamycin sensitivity [46,47]. Genes affecting rapamycin sensitivity are mainly involved in upregulation of TOR signalling, as gene deletions cause hypersensitivity to rapamycin. By contrast, our screening employed a ts mutant library in which cell proliferation is disrupted at the restrictive temperature, but is rescued by rapamycin. Rapamycin actions apparently compensate for these deletions and ts mutants. Genes identified in the present study seem to be required to balance TOR signalling for continuous cell proliferation (figure 5*e*) and raise the possibility of therapeutic applications for rapamycin. Notably, human orthologues of Erg10, Mis4 and Mrs6 are reportedly associated with beta-ketothiolase deficiency, Cornelia de Lange syndrome and intellectual disability, respectively [48–50]. In addition, Cut2/PTTG, a regulator of Cut1/ESPL1, is reportedly associated with cancer [51], while the SAPK cascade is a key therapeutic target of inflammatory disease [52,53]. Our results suggest the potential use of rapamycin to cure or ameliorate these diseases resulting from these genes.

## 4. Material and methods

### 4.1. Strains

A collection of 1014 ts mutant strains made by random mutagenesis was used [20]. To identify the mutation responsible for the phenotype, first, a plasmid set containing fragments of an *S. pombe* genomic library was introduced to each strain. Plasmids that rescued the ts phenotype were sequenced to identify suppressor genes. Second, if suppressor genes were definitive, tetrad analysis was conducted to identify the responsible gene. Or if several suppressor genes were identified, whole-genome sequencing was employed as follows. Mutant strains were backcrossed with WT to collect segregants showing the ts phenotype (ts^−^). DNA samples from several ts^−^ segregants were extracted, mixed equally and sequenced using a Genome Analyzer IIx sequencer (Illumina). Background mutations were eliminated and mutations common to all ts^−^ segregants, which were also identified previously among suppressor plasmids, were confirmed as the responsible genes. The other strain used in this study was h^−^ Δ*sty1::ura4^+^ ura4-D18* (KS1366) [21] for the Δ*sty1* study.

### 4.2. Screening method and spot test assays

Rough screening of all 1014 strains was conducted using an automated robot system (Biomek FX, Beckman Coulter). Cells were cultivated in YPD liquid media and adjusted to a concentration of 1 × 10^6^ cells ml^−1^, after which 3 µl were spotted on YPD agar plates with or without 0.1 µg ml^−1^ rapamycin (figure 1*a*). Plates were incubated at 36°C for 3 days, and colony formation was compared. Subsequent manual spot tests were conducted by cultivating cells to 1 × 10^7^ cells ml^−1^ and serially diluting them in five steps (10-fold dilution in each step). Then 5 µl of each dilution were spotted on new plates. Spotted plates were incubated at 36°C.

### 4.3. Nitrogen deprivation, cell number, viability assays and flow cytometry

Nitrogen deprivation was accomplished by switching the culture media of exponentially growing cells from EMM2 to EMM2-N (EMM2-N lacks NH_4_Cl) by vacuum filtration, as described previously [54]. Cell number was measured using a Multisizer3 coulter counter (Beckman Coulter), and cell viability was measured by plating 300 cells on YPD plates and determining the percentage of the number of colonies formed per plated cell. To measure the DNA content, FACS analysis was conducted using a FACSCalibur (Becton Dickinson).

### 4.4. Microscopy

For DAPI staining, cells were fixed with 2% glutaraldehyde for 10 min on ice, washed 3× with phosphate-buffered saline (PBS), and observed under a fluorescence microscope (Axioplan2, ZEISS) after mixing with DAPI (25 µg ml^−1^).

### 4.5. Metabolome analysis

WT and *Δsty1* samples in liquid EMM2 with DMSO or 200 nM rapamycin were incubated for 6 h at 36°C and 40 ml of 5 × 10^6^ cells ml^−1^ were harvested. Metabolome samples were prepared as described previously [55,56] and protein amount for each sample was measured using Direct Detect (Merck). Samples were spiked with two internal standards, PIPES and HEPES, corrected for protein quantity, extracted and separated by liquid chromatography on a ZIC-pHILIC column (Merck), and then measured using an LTQ Orbitrap mass spectrometer (Thermo Fisher Scientific). MZmine2 software was used for raw mass spectrum analysis [57]. From areas of detected peaks, principal components analyses were calculated and the two principal components were plotted using SIMCA P+ software (Sartorius Stedim) to show clear differentiation of the triplicates under each condition (figure 4*b*).

Among detected peaks, 95 metabolites were identified by comparing *m/z* values and retention times with authentic standards. Additionally, FGAM, FGAR and SAICAR were identified as previously explained [56].

## Supplementary Material

Fig. S1. Rough spot tests identified 62 candidate strains, the ts phenotype of which could be rescued by 0.1 µg/mL rapamycin.; Fig. S2. Manual spot tests of 62 selected candidates identified 45 strains with clear rescue at 36ºC (underlined).; Fig. S3. 19 strains with cut1 mutations showed a ts phenotype rescued by rapamycin.; Fig. S4. Rapamycin rescued the ts phenotype of sty1-989.;

## Supplementary Material

Table S1. 12 genes responsible for temperature sensitivity were determined.

## Data accessibility

The datasets supporting this article have been uploaded as part of the electronic supplementary material.
