# Supplementary material for: Genetic defects in SAPK signalling, chromatin regulation, vesicle transport and CoA-related lipid metabolism are rescued by rapamycin in fission yeast
Source: Open Biol. 2018 Mar 28;8(3):170261. doi: 10.1098/rsob.170261 (PMC5881033; doi:10.1098/rsob.170261)
Supplement: Fig. S1. Rough spot tests identified 62 candidate strains, the ts phenotype of which could be rescued by 0.1 µg/mL rapamycin.; Fig. S2. Manual spot tests of 62 selected candidates identified 45 strains with clear rescue at 36ºC (underlined).; Fig. S3. 19 strains with cut1 mutations showed a ts pheno [file rsob170261supp1.pdf]

Fig. S1

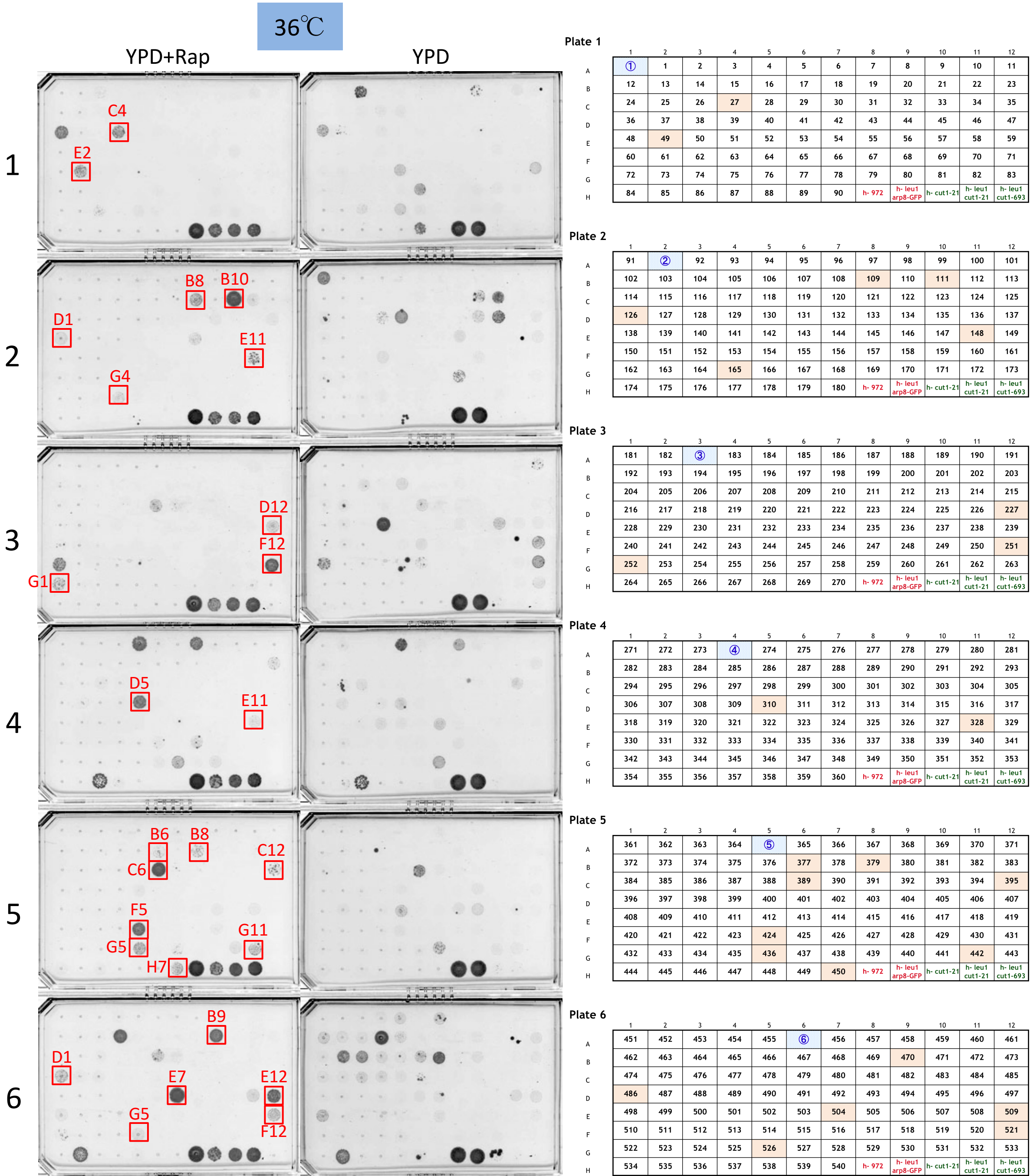

Plate 1

	1	2	3	4	5	6	7	8	9	10	11	12
A	①	1	2	3	4	5	6	7	8	9	10	11
B	12	13	14	15	16	17	18	19	20	21	22	23
C	24	25	26	27	28	29	30	31	32	33	34	35
D	36	37	38	39	40	41	42	43	44	45	46	47
E	48	49	50	51	52	53	54	55	56	57	58	59
F	60	61	62	63	64	65	66	67	68	69	70	71
G	72	73	74	75	76	77	78	79	80	81	82	83
H	84	85	86	87	88	89	90	h- 972	h- leu1 arp8-GFP	h- cut1-21	h- leu1 cut1-21	h- leu1 cut1-693

Plate 2

	1	2	3	4	5	6	7	8	9	10	11	12
A	91	②	92	93	94	95	96	97	98	99	100	101
B	102	103	104	105	106	107	108	109	110	111	112	113
C	114	115	116	117	118	119	120	121	122	123	124	125
D	126	127	128	129	130	131	132	133	134	135	136	137
E	138	139	140	141	142	143	144	145	146	147	148	149
F	150	151	152	153	154	155	156	157	158	159	160	161
G	162	163	164	165	166	167	168	169	170	171	172	173
H	174	175	176	177	178	179	180	h- 972	h- leu1 arp8-GFP	h- cut1-21	h- leu1 cut1-21	h- leu1 cut1-693

Plate 3

	1	2	3	4	5	6	7	8	9	10	11	12
A	181	182	③	183	184	185	186	187	188	189	190	191
B	192	193	194	195	196	197	198	199	200	201	202	203
C	204	205	206	207	208	209	210	211	212	213	214	215
D	216	217	218	219	220	221	222	223	224	225	226	227
E	228	229	230	231	232	233	234	235	236	237	238	239
F	240	241	242	243	244	245	246	247	248	249	250	251
G	252	253	254	255	256	257	258	259	260	261	262	263
H	264	265	266	267	268	269	270	h- 972	h- leu1 arp8-GFP	h- cut1-21	h- leu1 cut1-21	h- leu1 cut1-693

Plate 4

	1	2	3	4	5	6	7	8	9	10	11	12
A	271	272	273	④	274	275	276	277	278	279	280	281
B	282	283	284	285	286	287	288	289	290	291	292	293
C	294	295	296	297	298	299	300	301	302	303	304	305
D	306	307	308	309	310	311	312	313	314	315	316	317
E	318	319	320	321	322	323	324	325	326	327	328	329
F	330	331	332	333	334	335	336	337	338	339	340	341
G	342	343	344	345	346	347	348	349	350	351	352	353
H	354	355	356	357	358	359	360	h- 972	h- leu1 arp8-GFP	h- cut1-21	h- leu1 cut1-21	h- leu1 cut1-693

Plate 5

	1	2	3	4	5	6	7	8	9	10	11	12
A	361	362	363	364	⑤	365	366	367	368	369	370	371
B	372	373	374	375	376	377	378	379	380	381	382	383
C	384	385	386	387	388	389	390	391	392	393	394	395
D	396	397	398	399	400	401	402	403	404	405	406	407
E	408	409	410	411	412	413	414	415	416	417	418	419
F	420	421	422	423	424	425	426	427	428	429	430	431
G	432	433	434	435	436	437	438	439	440	441	442	443
H	444	445	446	447	448	449	450	h- 972	h- leu1 arp8-GFP	h- cut1-21	h- leu1 cut1-21	h- leu1 cut1-693

Plate 6

	1	2	3	4	5	6	7	8	9	10	11	12
A	451	452	453	454	455	⑥	456	457	458	459	460	461
B	462	463	464	465	466	467	468	469	470	471	472	473
C	474	475	476	477	478	479	480	481	482	483	484	485
D	486	487	488	489	490	491	492	493	494	495	496	497
E	498	499	500	501	502	503	504	505	506	507	508	509
F	510	511	512	513	514	515	516	517	518	519	520	521
G	522	523	524	525	526	527	528	529	530	531	532	533
H	534	535	536	537	538	539	540	h- 972	h- leu1 arp8-GFP	h- cut1-21	h- leu1 cut1-21	h- leu1 cut1-693

Fig. S1

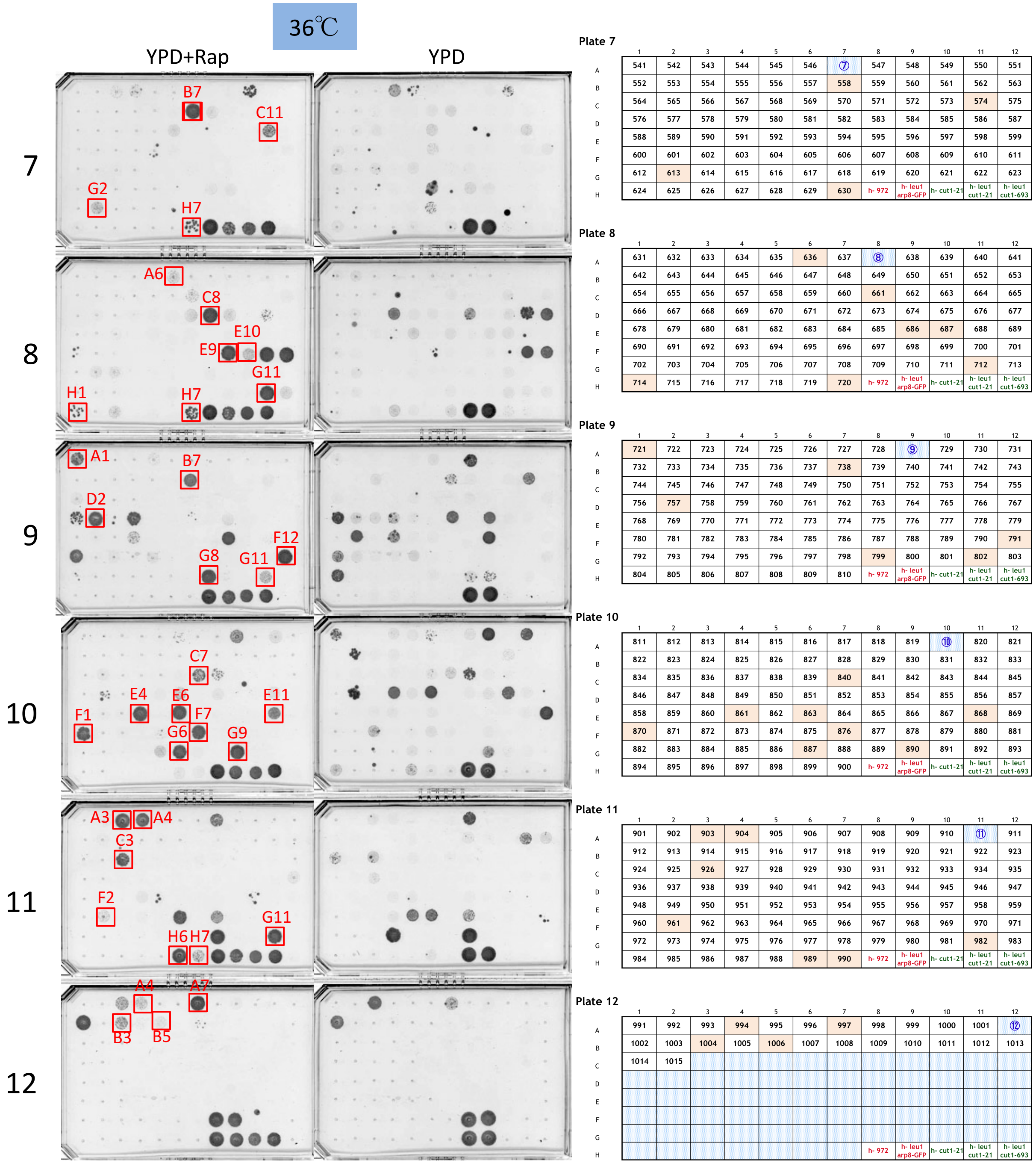

Plate 7

	1	2	3	4	5	6	7	8	9	10	11	12
A	541	542	543	544	545	546	⑦	547	548	549	550	551
B	552	553	554	555	556	557	558	559	560	561	562	563
C	564	565	566	567	568	569	570	571	572	573	574	575
D	576	577	578	579	580	581	582	583	584	585	586	587
E	588	589	590	591	592	593	594	595	596	597	598	599
F	600	601	602	603	604	605	606	607	608	609	610	611
G	612	613	614	615	616	617	618	619	620	621	622	623
H	624	625	626	627	628	629	630	h- 972	h- leu1 arp8-GFP	h- cut1-21	h- leu1 cut1-21	h- leu1 cut1-693

Plate 8

	1	2	3	4	5	6	7	8	9	10	11	12
A	631	632	633	634	635	636	637	⑧	638	639	640	641
B	642	643	644	645	646	647	648	649	650	651	652	653
C	654	655	656	657	658	659	660	661	662	663	664	665
D	666	667	668	669	670	671	672	673	674	675	676	677
E	678	679	680	681	682	683	684	685	686	687	688	689
F	690	691	692	693	694	695	696	697	698	699	700	701
G	702	703	704	705	706	707	708	709	710	711	712	713
H	714	715	716	717	718	719	720	h- 972	h- leu1 arp8-GFP	h- cut1-21	h- leu1 cut1-21	h- leu1 cut1-693

Plate 9

	1	2	3	4	5	6	7	8	9	10	11	12
A	721	722	723	724	725	726	727	728	⑨	729	730	731
B	732	733	734	735	736	737	738	739	740	741	742	743
C	744	745	746	747	748	749	750	751	752	753	754	755
D	756	757	758	759	760	761	762	763	764	765	766	767
E	768	769	770	771	772	773	774	775	776	777	778	779
F	780	781	782	783	784	785	786	787	788	789	790	791
G	792	793	794	795	796	797	798	799	800	801	802	803
H	804	805	806	807	808	809	810	h- 972	h- leu1 arp8-GFP	h- cut1-21	h- leu1 cut1-21	h- leu1 cut1-693

Plate 10

	1	2	3	4	5	6	7	8	9	10	11	12
A	811	812	813	814	815	816	817	818	819	⑩	820	821
B	822	823	824	825	826	827	828	829	830	831	832	833
C	834	835	836	837	838	839	840	841	842	843	844	845
D	846	847	848	849	850	851	852	853	854	855	856	857
E	858	859	860	861	862	863	864	865	866	867	868	869
F	870	871	872	873	874	875	876	877	878	879	880	881
G	882	883	884	885	886	887	888	889	890	891	892	893
H	894	895	896	897	898	899	900	h- 972	h- leu1 arp8-GFP	h- cut1-21	h- leu1 cut1-21	h- leu1 cut1-693

Plate 11

	1	2	3	4	5	6	7	8	9	10	11	12
A	901	902	903	904	905	906	907	908	909	910	⑪	911
B	912	913	914	915	916	917	918	919	920	921	922	923
C	924	925	926	927	928	929	930	931	932	933	934	935
D	936	937	938	939	940	941	942	943	944	945	946	947
E	948	949	950	951	952	953	954	955	956	957	958	959
F	960	961	962	963	964	965	966	967	968	969	970	971
G	972	973	974	975	976	977	978	979	980	981	982	983
H	984	985	986	987	988	989	990	h- 972	h- leu1 arp8-GFP	h- cut1-21	h- leu1 cut1-21	h- leu1 cut1-693

Plate 12

	1	2	3	4	5	6	7	8	9	10	11	12
A	991	992	993	994	995	996	997	998	999	1000	1001	⑫
B	1002	1003	1004	1005	1006	1007	1008	1009	1010	1011	1012	1013
C	1014	1015										
D												
E												
F												
G												
H								h- 972	h- leu1 arp8-GFP	h- cut1-21	h- leu1 cut1-21	h- leu1 cut1-693

Fig. S2

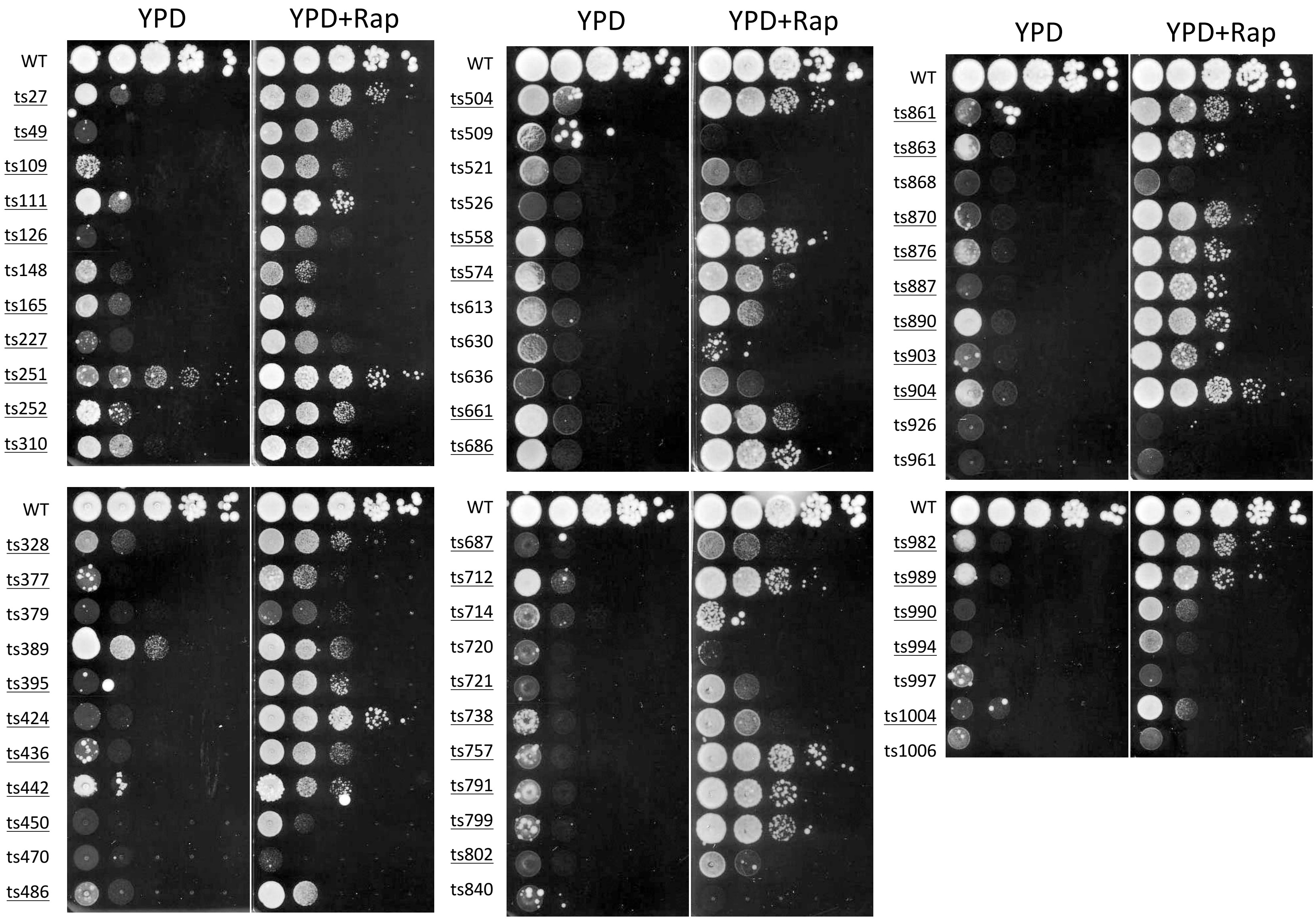
YPD
YPD+Rap
WT
ts27
ts49
ts109
ts111
ts126
ts148
ts165
ts227
ts251
ts252
ts310
YPD
YPD+Rap
WT
ts504
ts509
ts521
ts526
ts558
ts574
ts613
ts630
ts636
ts661
ts686
YPD
YPD+Rap
WT
ts861
ts863
ts868
ts870
ts876
ts887
ts890
ts903
ts904
ts926
ts961
WT
ts328
ts377
ts379
ts389
ts395
ts424
ts436
ts442
ts450
ts470
ts486
WT
ts687
ts712
ts714
ts720
ts721
ts738
ts757
ts791
ts799
ts802
ts840
WT
ts982
ts989
ts990
ts994
ts997
ts1004
ts1006

Fig. S3

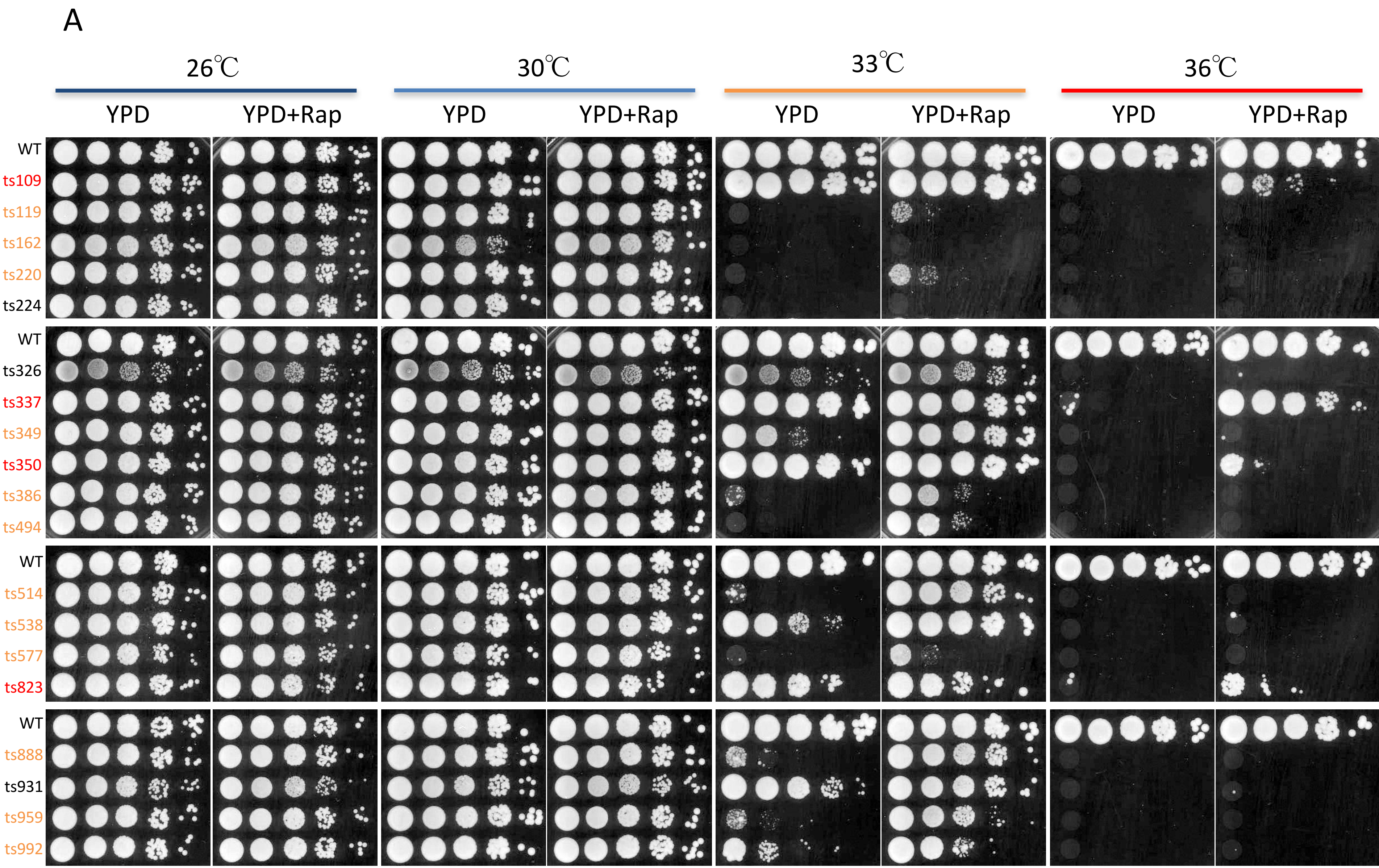
A
26℃
30℃
33℃
36℃
YPD
YPD+Rap
YPD
YPD+Rap
YPD
YPD+Rap
YPD
YPD+Rap
WT
ts109
ts119
ts162
ts220
ts224
WT
ts326
ts337
ts349
ts350
ts386
ts494
WT
ts514
ts538
ts577
ts823
WT
ts888
ts931
ts959
ts992

Fig. S3

B

cut1 mutants	Base change	A.A. change
109	G3597A	G1160E
119	G2493A	E805K
162	G2937A	G940D
220	G5234A	G1706R
224	C5195T	L1693F
326	G5066A	G1650S
337	C5567T	P1817S
349	G4149A, G5139A	S1344N, G1674D
350	A2873G	T919A
386	G3098A	E994K
494	A5094G	D1659G
514	A3102G	H995R
538	C5160T	A1681V
577	G5417A	D1767N
823	T82C	C28R
888	G5139A	G1674D
931	G5139A	G1674D
959	T3308C	W1064R
992	G2493A	E805K

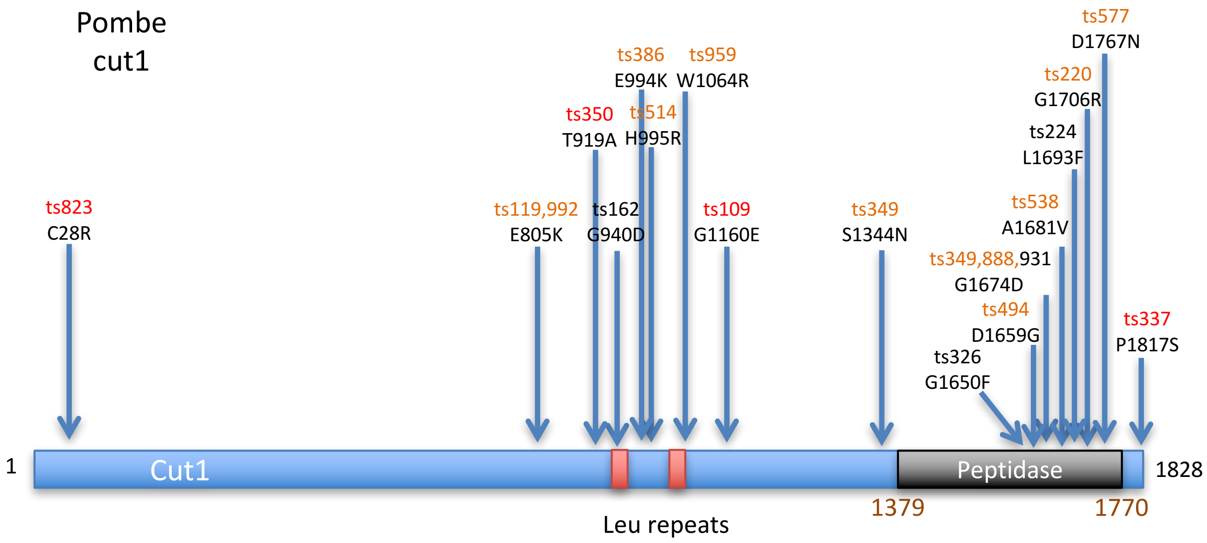

Fig. S4

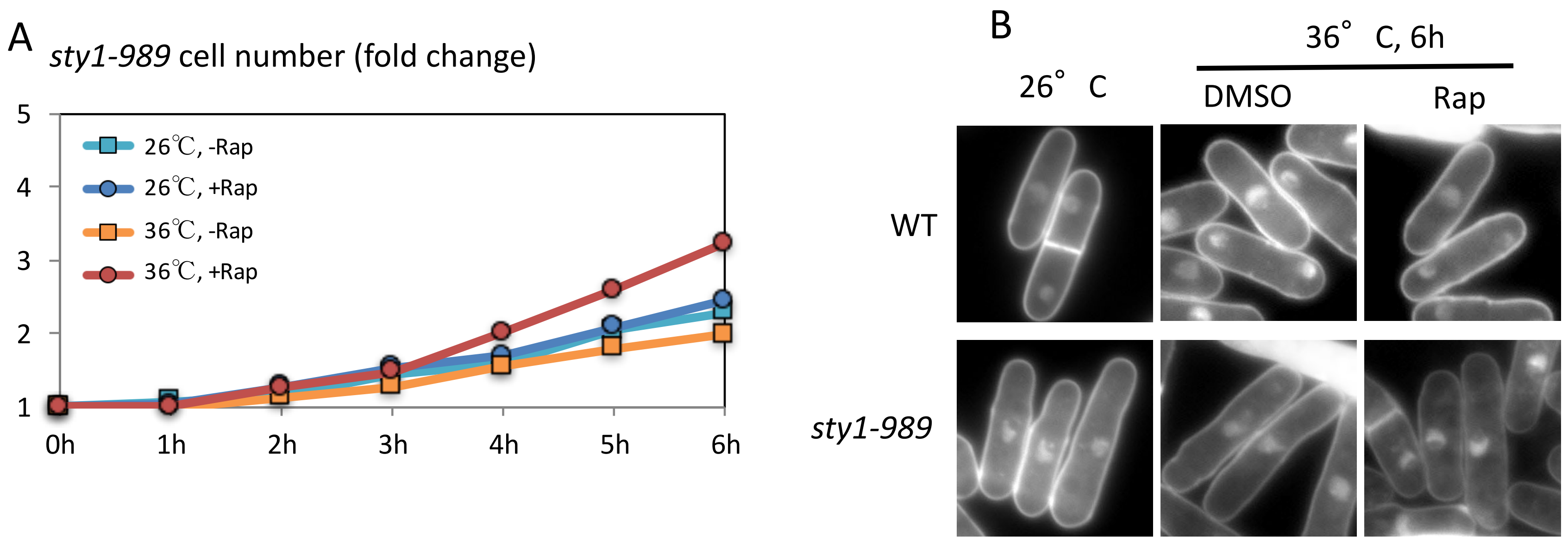

A
sty1-989 cell number (fold change)
5
4
3
2
1
0h
1h
2h
3h
4h
5h
6h
26℃, -Rap
26℃, +Rap
36℃, -Rap
36℃, +Rap
B
36° C, 6h
26° C
DMSO
Rap
WT
sty1-989
